# Regenerative Engineering Animal Models for Knee Osteoarthritis

**DOI:** 10.1007/s40883-021-00225-y

**Published:** 2021-07-29

**Authors:** Caldon Jayson Esdaille, Chinedu Cletus Ude, Cato T. Laurencin

**Affiliations:** 1 Washington, USA; 2 Connecticut Convergence Institute for Translation in Regenerative Engineering, University of Connecticut Health Center, 263 Farmington Avenue, Farmington, CT, USA; 3 Raymond and Beverly Sackler Center for Biomedical, Biological, Physical and Engineering Sciences, University of Connecticut Health, Farmington, CT, USA; 4 Department of Orthopaedic Surgery, University of Connecticut Health, Farmington, CT, USA; 5 Department of Chemical and Biomolecular Engineering, University of Connecticut, Storrs, CT, USA; 6 Department of Biomedical Engineering, University of Connecticut, Storrs, CT, USA; 7 Department of Materials Science and Engineering, University of Connecticut, Storrs, CT, USA; 8 Institute of Materials Science, University of Connecticut, Storrs, CT, USA; 9 Department of Craniofacial Sciences, School of Dental Medicine, University of Connecticut Health, Farmington, CT, USA

**Keywords:** Regenerative engineering, Osteoarthritis, Animal models, Preclinical trials, Translation

## Abstract

Osteoarthritis (OA) of the knee is the most common synovial joint disorder worldwide, with a growing incidence due to increasing rates of obesity and an aging population. A significant amount of research is currently being conducted to further our understanding of the pathophysiology of knee osteoarthritis to design less invasive and more effective treatment options once conservative management has failed. Regenerative engineering techniques have shown promising preclinical results in treating OA due to their innovative approaches and have emerged as a popular area of study. To investigate these therapeutics, animal models of OA have been used in preclinical trials. There are various mechanisms by which OA can be induced in the knee/stifle of animals that are classified by the etiology of the OA that they are designed to recapitulate. Thus, it is essential to utilize the correct animal model in studies that are investigating regenerative engineering techniques for proper translation of efficacy into clinical trials. This review discusses the various animal models of OA that may be used in preclinical regenerative engineering trials and the corresponding classification system.

## Lay Summary

Osteoarthritis (OA) of the knee is the most common synovial joint disease worldwide, with high rates of occurrence due to an increase in obesity and an aging population. A great deal of research is currently underway to further our understanding of the causes of osteoarthritis, to design more effective treatments. The emergence of regenerative engineering has provided physicians and investigators with unique opportunities to join ideas in tackling human diseases such as OA. Once the concept is proven to work, the initial procedure for the evaluation of a treatment solution begins with an animal model. Thus, it is essential to utilize a suitable animal model that reflects the particular ailment in regenerative engineering studies for proper translation to human patients as each model has associated advantages and disadvantages. There are various ways by which OA can occur in the knee joint, which are classified according to the particular cause of the OA. This review discusses the various animal models of OA that may be used in preclinical regenerative engineering investigations and the corresponding classification system.

## Introduction

Regenerative engineering has recently emerged as a solution for complex clinical challenges with tremendous growth and expansion of the field in the last 25 years. By utilizing the convergence of the disciplines of advanced material science, stem cell science, physics, developmental biology, and clinical translation, the field of regenerative engineering has shown very promising results in its ability to harness the body’s healing and regenerative abilities [1–3]. One of the most prevalent diseases that has been investigated in regenerative engineering studies is osteoarthritis (OA). Ongoing research in biomaterials and stem cell-based therapies has shown promising results in preclinical and clinical trials demonstrating that cartilage and bone regeneration could provide a long-term solution to OA and prevent progression to end-stage disease [4–11].

OA is the most common form of arthritis and synovial joint disorders worldwide. It is characterized by chronic pain of the affected joint with varying symptoms and severity. OA can be defined clinically by symptoms such as pain or swelling in the joint, structural pathology, or a combination of the two [12, 13]. Approximately 14 million people in the USA suffer from symptomatic OA, with the knee being the most commonly involved joint [14, 15]. Among these cases, it is estimated that more than 3 million persons are minorities with a higher incidence in individuals between the ages of 45 and 65. With the prevalence of OA increasing yearly due to the aging population and growing rates of obesity, the development of treatment regimens to prevent end-stage disease is of utmost importance. Conservative treatments are usually unable to provide long-term relief of symptoms in patients with end-stage disease, resulting in consideration of a total knee arthroplasty (TKA) as a final treatment [12, 15]. Although arthroplasty has had high rates of success, it can be an expensive procedure that may place a financial burden on a population of patients. In addition, all patients are not surgical candidates due to existing comorbidities [16, 17].

Many regenerative engineering preclinical trials incorporate the use of OA animal models to further investigate the efficacy of therapies. Due to limitations such as variation of symptoms and onset in patients as well as ethical issues that surround human clinical trials, animal trials are widely utilized [18, 19]. In addition, while in vitro studies may be useful for proof-of-concept studies, they do not simulate the characteristics of the actual joint space, biomechanics, or surrounding tissues, which compels the use of an animal model for the advancement of an investigation involving regenerative medicine. OA animal models have several different classifications based on their method of induction, providing investigators with approaches to study OA pathogenesis and design therapeutics based on the pathophysiology [18]. This paper will provide a comprehensive overview of knee OA animal models which can be used for regenerative engineering investigations along with highlights and findings of studies in which certain models have been utilized. A brief summarization of the pathophysiology of OA will also be included.

## Osteoarthritis Pathophysiology

**Fig. 1:**
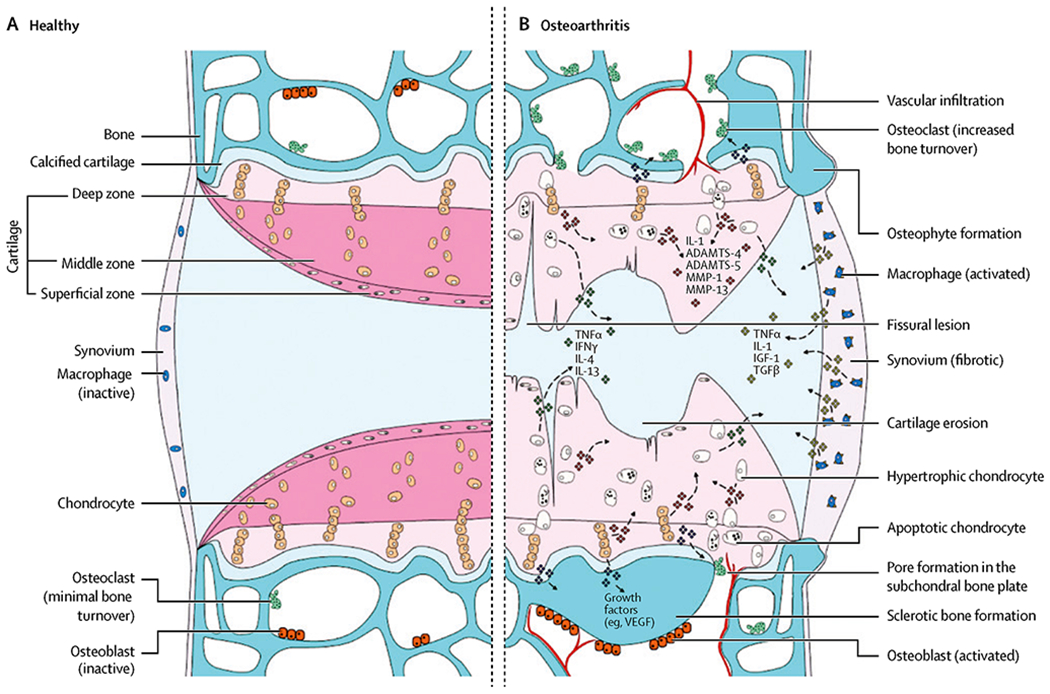
Comparison of normal (**A**) and osteoarthritic (**B**) joints. **A** Normal synovial joint and structures. **B** The signaling pathways and structural changes that occur as osteoarthritis develops within a diseased joint. *ADAMTS*, a disintegrin and metalloproteinase with thrombospondin-like motifs; *IL*, interleukin; *MMP*, matrix metalloproteinase; *TNF*, tumor necrosis factor; *IFN*, interferon; *IGF*, insulin-like growth factor; *TGF*, transforming growth factor; *VEGF*, vascular endothelial growth factor (Glyn-Jones et al. [12])

Progressive loss and destruction of articular cartilage, thickening of subchondral bone, formation of osteophytes, inflammation of the synovium, and degeneration of ligaments and menisci all contribute to the pathology of knee OA [12, 20]. The etiology of these pathologies is multifactorial due to a combination of injury, obesity, aging, and genetics on cartilage, subchondral bone, and synovium [12]. The molecular pathways that may contribute to the development of OA are reviewed in Fig. 1. Inflammation is a common factor among most mechanisms of OA as the entire synovial joint, menisci, articular cartilage, synovium, and subchondral bone can be affected [20, 21]. In a healthy individual, the meniscus is fibrocartilage composed of type I collagen surrounded by proteoglycans in the extracellular matrix (ECM) that serve as shock absorbers. This fibrocartilage also provides weight-bearing joint support, lubrication, and congruity [22–24]. Articular cartilage is composed of type II collagen and provides an ideal surface for the movement of the synovial joint. Calcifications to this cartilage may also impede the interactions between bone and cartilage [22, 25]. The synovial fluid, which is regulated by the synovial membrane, contains lubricin, hyaluronic acid, fibroblasts, and macrophages; lubricates the joint surface; and provides nourishment for the articular cartilage [26].

Aging is the most common risk factor for OA due to the changes in articular chondrocytes. The ECM of articular cartilage is composed of type II collagen as the main structural protein along with proteoglycans and chondrocytes [22]. The role of the chondrocyte is to maintain the structure of cartilage by the production of extracellular matrix components. As chondrocytes age, they develop a senescent phenotype, which impairs the ability to respond to mechanical and inflammatory insults [20, 27]. Excessive joint loading is the main mechanism of OA in obese individuals as structural joint damage follows as a result of altered biomechanics in everyday activities. Both weight-bearing and non-weight-bearing areas of joints are affected which leads to the progression of OA [28]. The impact of body mass index (BMI) on osteoarthritis of the knee occurs in a dose-dependent manner as a 5-unit increase in BMI has been observed to have a 35% increased risk of knee OA [29, 30].

Post-traumatic OA (PTOA) is most commonly seen in younger adults after damage to the anterior cruciate ligament, menisci, or an intra-articular fracture which can also negatively affect joint biomechanics and lead to long-term degenerative changes. Long-term follow-up studies demonstrated that 41–51% of patients developed osteoarthritis 12–14 years after their initial injury [31]. Injuries result in chondrocyte death, bone bruising, hemarthrosis, and the release of inflammatory mediators in the acute post-injury period [32]. Previous studies have analyzed synovial fluid in patients with traumatic anterior cruciate ligament (ACL) tears and found high levels of interleukin-1 alpha (IL-1α), IL-6, IL-8, and tumor necrosis factor-alpha (TNF-α). Levels were highest at days 0–1 post-injury but remained elevated when compared to non-injured controls [33]. Trauma may also lead to microfractures of the articular cartilage which can produce “wear and tear particles.” The presence of these particles can overwhelm the ability of chondrocytes to maintain normal homeostasis between synthesis and degradation of extracellular matrix components by synovial macrophages [34]. These particles may become mediators of inflammation and lead to the release of proinflammatory cytokines from the synovium such as TNF-α, IL-1, IL-4, IL-6, IL-9, and IL-13 [35, 36]. Death of chondrocytes via apoptosis is also a major mechanism for the progression of OA as IL-1, TNF-α, and nitric oxide (NO) induce apoptosis of chondrocytes. Hypertrophy of the chondrocytes occurs subsequently and diminishes the ability to produce a new cartilage matrix [37]. Subchondral bone also undergoes abnormal remodeling and sclerosis in late-stage OA, producing subchondral cysts, calcifications, and osteophytes to maintain joint stability [38]. Osteophytes are fibrocartilage-capped bony outgrowths derived from precursor cells in the periosteum that are induced by transforming growth factor-beta (TGF-β). The outgrowths may be a source of pain and loss of function by nerve compression, limiting joint mobility and obstruction of tissues [39].

## Osteoarthritis Animal Model Classifications

**Fig. 2:**
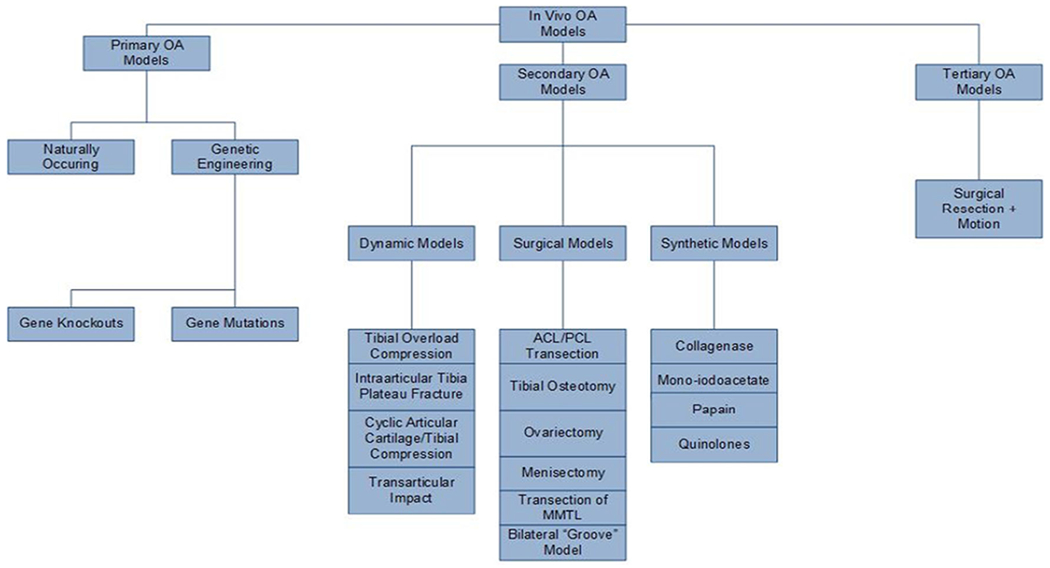
Proposed classification of OA animal models in vivo. *ACL*, anterior cruciate ligament; *PCL*, posterior cruciate ligament; *MMTL*, medial meniscotibial ligament

OA animal models are classically categorized based on their method of induction of osteoarthritis, which also reflects the clinical classification of OA based on disease etiology as defined by the American College of Rheumatology [18, 40]. Primary OA occurs as a result of degenerative changes in the knee joint, commonly thought of as a naturally occurring phenomenon. Secondary OA usually occurs in association with several risk factors, such as trauma, injury, or metabolic disease that lead to the development of a degenerative joint disease or PTOA [18, 41]. These animal models are usually induced by invasive surgical methods or noninvasive mechanical stimuli (Fig. 2). However, there are some limitations for the traditional classifications as these models are not all-inclusive of the different etiologies for OA. The tertiary animal model classification that we are proposing in this review seeks to provide another modality of inducing an OA in vivo animal model providing another option and flexibility for translation into clinical trials. A tertiary animal model combines a secondary method of osteoarthritis with a mechanism to accelerate disease progression. Surgical induction of OA would occur initially, followed by a routine and monitored exercise regime to simulate a severe multifocal degenerative joint disorder of the knee that was operated on. This will produce a variant of PTOA that is typical of OA in populations who have had untreated or undetected trauma in combination with overuse of the affected joint.

## Primary Osteoarthritis Models

The strength of primary or spontaneous models of osteoarthritis is that they closely resemble the natural progression of human primary OA. In certain animal models including mice, guinea pigs, dogs, rabbits, and horses, OA is a slowly progressive condition with an insidious onset [42, 43]. Although time consuming in comparison to surgically induced models, the pathophysiology is comparable to humans who develop OA in a nontraumatic process with an insidious onset. The subcategories for primary OA models are naturally occurring and genetically modified, both with specific advantages and disadvantages [44]. However, the main disadvantage of primary OA models is the time required for the induction of OA. Although it produces a model that is quite representative of human pathophysiology, most researchers may elect to utilize another method with more rapid induction of OA.

### Animal Models with Naturally Occurring OA

Mice, rabbits, dogs, horses, and certain strains of guinea pigs are the most widely used animals for naturally occurring OA. The albino Dunkin Hartley or Hartley guinea pigs have been described throughout literature as a great representation of human primary OA due to histopathological similarities between both species [45, 46]. In addition, the rapid skeletal maturity compared to other spontaneous animal models poses a great advantage among naturally induced OA models [47]. Guinea pigs also have a varus alignment of the knees, which places increased load in the medial compartment and predisposes them to medial compartment OA, a very common presentation among humans [46]. The relative ease of handling of these animals and the joint size which allows for sufficient tissue and synovial fluid collection and analysis present other advantages [48].

Particular strains of mice have been identified as having a genetic predisposition to developing spontaneous OA. Mice strains, *STR/ort*, and *C57BL/6* are used for studies involving OA pathogenesis and therapeutics. Studies have reported a high incidence of knee OA in these mice as early as 18 weeks of age. Other mice strains, such as CBA, have been identified as resistant to the development of OA [44, 49].

### Genetically Modified Animal Models

Genetic engineering has allowed scientists to explore gene knockouts and knock-ins to determine genetic factors involved in OA pathogenesis [18]. For example, *Col2a1* knockout mice have a higher incidence (60–90%) of natural OA than wild type [50]. Mice with a collagen type IX alpha 1 gene inactivation, *Col9a1* (−/−), have been used to study and characterize the role of collagen type IX in the development of OA [51]. Although genetic alterations and engineering have played a critical role in the understanding of disease pathogenesis, it is difficult to incorporate therapeutic options that target these genetic abnormalities in animal models. Genetically modified models have been particularly useful in osteoarthritis studies that are investigating the pathogenesis and impact of genetics on the development of OA and are less prevalent in studies investigating therapeutic intervention [52]. Other models have been reported to be more accurate in the study of the efficacy of therapeutic interventions [52]. As a result, most investigators will opt to use a more efficient animal model in their investigation due to these challenges and limitations.

## Secondary Osteoarthritis Animal Models

Secondary OA encompasses OA that has occurred in conjunction with specific risk factors that include but are not limited to trauma, metabolic bone disease, and bone and joint disorders such as rheumatoid arthritis and calcium deposition. However, post-traumatic OA (PTOA) models are the most widely studied and utilized category of secondary OA models [53]. We propose that post-traumatic OA models can be further divided into two subcategories: (1) surgically induced models where there is a direct injury to the joint using an invasive method and (2) dynamic models where OA is induced by using a noninvasive procedure involving a single or repetitive mechanical stimulus to produce an insult. Synthetic or chemically induced models are another subcategory of secondary osteoarthritis that does not fall under the PTOA category. Synthetic models are the result of a chemical induction of OA which occurs within the knee joint, secondary to a chemical reaction.

### Surgically Induced Animal Models

Surgical-induced animal models utilize invasive procedures to study the disease pathology of PTOA of the knee. The rapid induction of OA and severe reproducible lesions allow studies that utilize these models to have a shorter experimental time frame which poses a great advantage for this model [18, 44]. Articular cartilage and the meniscus are the two main tissues affected in PTOA. As a result, most of the surgically induced animal models focus on creating a mechanical disturbance of these tissues through alteration of tissues within the joint such as cruciate and/or collateral ligaments to promote the onset of disease [54]. Surgical animal models have been largely successful and, as a result, have been incorporated into several regenerative engineering studies. However, this rapid induction of disease may prove to be difficult to use in studies involving regenerative therapy, which targets the mechanism of early stages of OA. In addition, surgical methods largely depend on the sterile nature of the procedure and the skills of the surgeon. Any infection, inflammatory changes, or mistakes in surgery can alter the results, which may make reproducibility difficult between animal models [18].

**Fig. 3:**
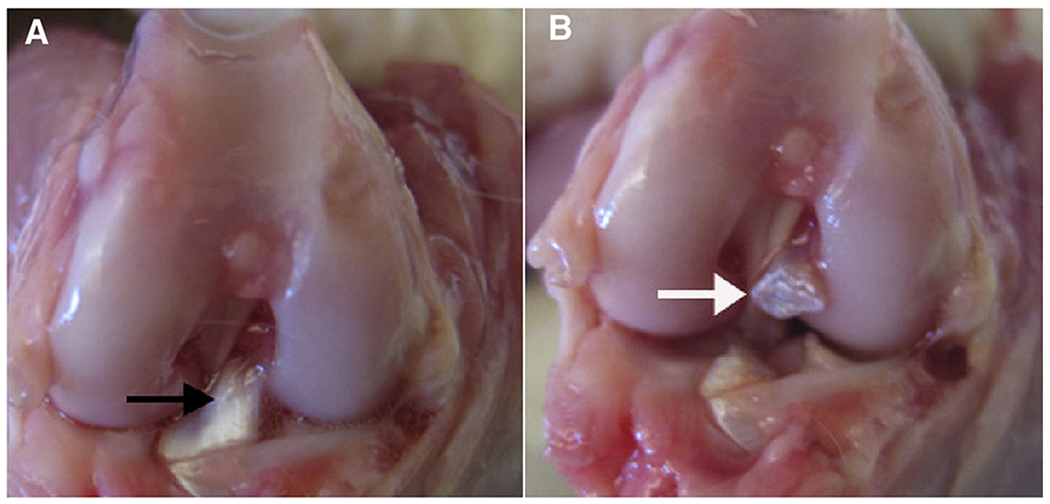
Gross images of a rabbit knee before (**A**) and after (**B**) transection of the ACL. **A** The black arrow indicates intact ACL. **B** The white arrow indicates the transected ACL (Lozano et al [55])

**Fig. 4:**
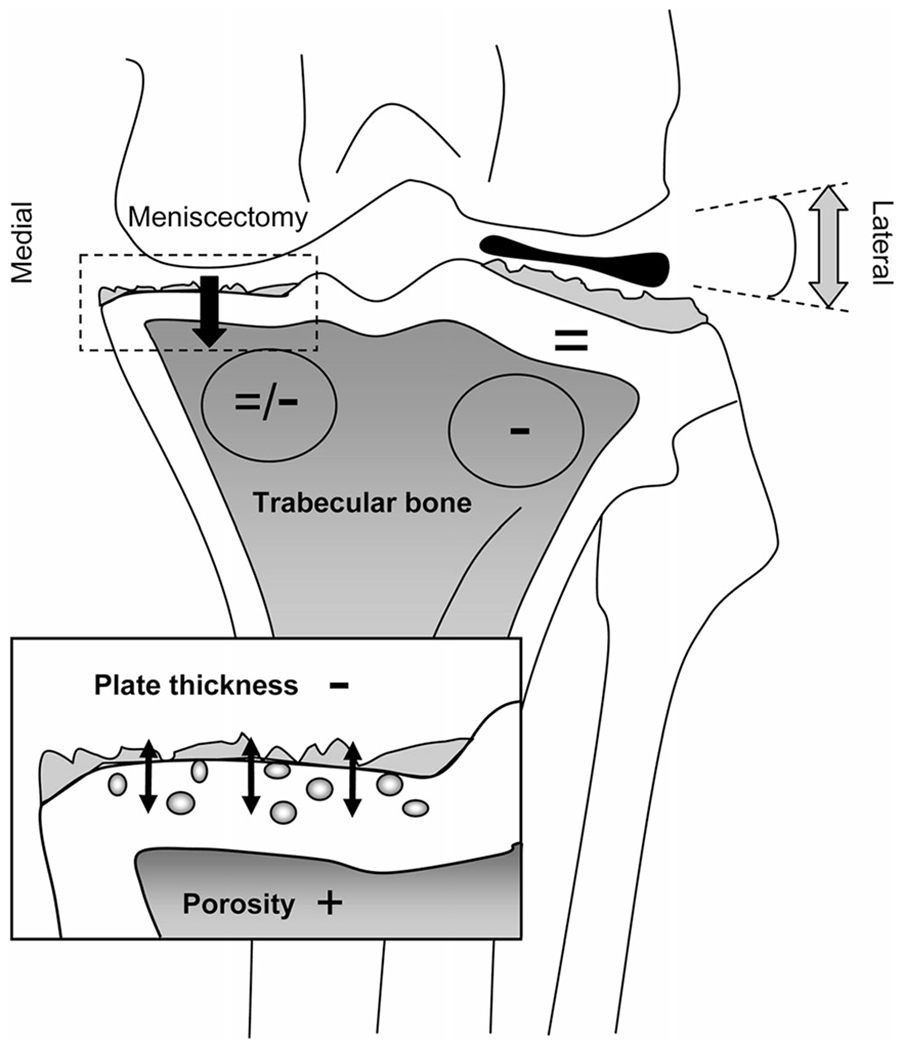
Schematic overview of changes in the ACLT-Meniscectomy model. Altered biomechanics due to post-surgical changes produces cartilage damage in both the medial and lateral compartments, but more severe osteoarthritic changes are noted on the medial compartment where the medial meniscus was removed. In the medial compartment, thinning and increased porosity of the subchondral plate are noted along with cartilage degeneration (inset). In the lateral compartment, trabecular bone decreases (−) indicating unloading either due to total paw unloading or locally due to the varus angle (arrows). Trabecular changes are not noted in the medial compartment (=/−) where the medial meniscus was removed (Intema et al. [58])

Anterior cruciate ligament transection (ACLT) is the most commonly utilized surgical method of inducing OA [18, 44, 54]. Injury to the ACL causes destabilization of the knee joint, which eliminates restraint to anterior translocation. This altered pattern of joint loading mechanics will lead to accelerated articular cartilage degeneration and the development of lesions that are comparable to OA in humans (Fig. 3) [54–56]. This method of induction of OA lesions has been advantageous in the study of pharmaceutical delivery due to its slower induction when compared to other surgically induced methods. Thus, this model may provide more appropriate clinical translatability among other invasive methods [57]. Some models also employ simultaneous transection of the posterior cruciate ligament (PCL), meniscus (Fig. 4), or medial and/or lateral collateral ligaments to study various grades of PTOA, whose pathogenesis is similar to humans who have had a traumatic injury without surgical repair or therapy [58, 59]. From an anatomical standpoint, goats, sheep, and cows can be used in this model due to the large size of their stifle, which allows for reproducible results between models. More specifically, in-depth studies of goat stifle have revealed that the anatomy is closest to the human knee [42, 60].

Various surgical manipulations of the meniscus can induce OA in animal models as changes in the meniscus morphology can produce osteoarthritic joints. Destabilization of the medial meniscus (DMM) in rodent models incorporates sectioning of the medial meniscotibial ligament (MMTL). The MMTL anchors the medial meniscus to the tibial plateau; disruption of the ligament results in destabilization of the medial meniscus causing it to be translocated medially [61]. This decreases the area for weight-bearing forces causing increased mechanical stress at the tibial plateau accelerating the progression to OA [44, 61]. A direct transection of the medial meniscus by removing the medial collateral ligaments to expose the meniscus can also produce a rapidly progressing OA variant within approximately 3 weeks in rats. The alteration is similar morphologically to osteoarthritic human joints that occur secondary to a medial meniscus tear. In humans, these osteoarthritic changes can be visualized radiographically 20 years following the meniscal injury [42, 44].

In rabbits, a partial meniscectomy can be performed on the medial or lateral meniscus with different osteoarthritic outcomes that are specific to the area removed. Rabbits are known to have high loading pressures in the lateral compartment of the joint [42]. This produces a more rapidly progressing OA with resection of the lateral meniscal when compared to rodents undergoing a similar procedure. In addition, more severe OA in rabbits is more commonly seen after a partial lateral meniscectomy when compared to a partial medial meniscectomy in the same species [42]. In humans, medial loading within the knee varies based on valgus or varus alignment. Medial degeneration is much more commonly observed clinically and radiographically in patients with OA [62]. Thus, animal models with medial degeneration are more representative of human pathology and may be more suitable for regenerative engineering therapeutics. The beagle is a commonly used animal in dog models as half of the meniscus can be transected without alterations of the collateral ligament. Dogs also load on the medial aspect of their stifle, which leads to more severe medial degeneration of the knee/stifle [42].

Estrogen deficiency in postmenopausal women increases the risk of OA; thus, ovariectomized female rats have been used for inducing OA [63]. Although initially used for osteoporosis animal studies, ovariectomies have shown mild osteoarthritic lesions of the stifle as early as 9 weeks post-surgery [64–66]. Ovariectomized New Zealand rabbits, mice, rats, guinea pigs, and sheep have been used to study OA. Since the mechanism of OA secondary to estrogen deficiency is still under investigation, many studies have used this model to look at pathological pathways in the development of OA. However, the study of a regenerative intervention is possible [18]. One model that has also emerged recently is the tibial osteotomy in mice. In this model, the tibia is surgically adjusted to a varus or valgus alignment altering the mechanical loading on the medial or lateral side of the knee joint [67]. This model avoids intra-articular surgery which poses the risk of damage to the intra-articular tissue and joints. Although this is a surgical method of inducing OA, it may potentially be used to investigate primary OA. This model is analogous to human primary OA where misalignment of the joint in a varus or valgus angle leads to OA without any internal damage to the joint [67].

Regenerative engineering studies that employ stem cell science commonly use surgical methods with damage to multiple tissues to assess the healing and regeneration of cartilage. Agung et al. [68] demonstrated that intra-articular injections of bone marrow-derived mesenchymal stem cells (BMSCs) into rats with resection of the ACL and medial meniscus showed mobilization of BMSCs to the injured site and tissue regeneration with ECM synthesis around the site of injury. In another study, Faqeh et al. [69] demonstrated that a single intra-articular dose of autologous BMSCs cultured in different media into a sheep model where OA was induced by ACL resection and total medial meniscectomy was able to diminish the progression of osteoarthritic lesions in the menisci and articular cartilage when compared to control groups. Adipose-derived stem cells (ADSCs) have also been studied using ACLT in adult rabbit models showing significant improvement in the quality of cartilage gross appearance and on histology by Mankin scoring at 20 weeks following surgery when compared to OA surgery [70]. Fernandez-Pernas et al. [71] also investigated CD105^+^ human synovial membrane-derived MSCs for cartilage regeneration but used adult Rhesus monkeys with a surgically induced 4-mm cartilage defect in the zone of maximum loading within the knee for their osteoarthritis model. Results revealed that MSCs were recruited to the injured joint directly from the bloodstream and injected cells remained within the joint space [71].

The efficacy of lyophilized implantable hyaluronic acid (HA) scaffolds and injectable HA hydrogels in the presence of corticosteroids for regeneration of articular cartilage has also been investigated using OA induced by ACL transection in rats. As HA is a major component in the ECM and cartilage tissue, intra-articular injections of HA are often used in conservative management of OA [41]. The investigators found that after 10 weeks, HA scaffolds and hydrogels both demonstrated repair to damaged articular cartilage with no additional benefit once corticosteroids were incorporated [72]. A similar study was conducted by Desano et al., who placed a hyaluronan-based scaffold, Hyaff®-11, seeded with autologous articular chondrocytes in early cartilage lesions in a rabbit model status post-ACLT. Histological analysis at 3 months and 6 months demonstrated OA lesions were significantly improved between groups treated with Hyaff®-11 seeded with chondrocytes when compared to just HA [73]. Grigolo et al. [74] further investigated Hyaff®-11 by seeding it with BMSCs and transplanting it into rabbit models 8 weeks after ACLT. Grossly, lesions were noted on the lateral and medial femoral condyles of controls with significant regeneration of cartilage in groups treated with scaffolds containing MSCs when compared to the hyaluronan-based scaffold alone. Overall, implantable scaffolds containing HA may be useful for articular cartilage regeneration, but corticosteroids although useful for the management of acute symptoms should not be used in conjunction.

### Dynamic Animal Models

Dynamic OA animal models are noninvasive and rely on a single or repetitive external insult to produce an osteoarthritic defect within the stifle or knee joint of the animal. The most common dynamic OA models used in mice are intra-articular tibial plateau fracture, cyclic AC tibial compression, and ACL rupture via compression overload [75, 76]. The fracture to the intra-articular tibial plateau is modeled after high-energy impact injuries such as a motor vehicle collision (MVC). In this model, the mouse joint is flexed and loaded onto a triangular cradle while a wedge-shaped indenter is mounted and used to apply a compressive load to the tibia, resulting in an articular fracture [18, 44, 77]. The amount of force applied to deliver controlled loads and displacements can be adjusted based on the desired result. Intra-articular tibial plateau fractures are one of the most common causes of PTOA in humans, especially after an MVC, which makes this model ideal for the investigation of regenerative therapeutics [78, 79].

Axial tibial loading has been established in the investigation of adaptive responses of cortical and trabecular bone to mechanical loading [44, 80]. An axial load is applied to the stifle of mice, leading to anterior displacement of the tibia relative to the femur. The load is applied to the tibia, in a cyclic manner or a one-time load, through the knee and ankle joints. Although one event is sufficient to produce an injury to the articular cartilage through a mechanoadaptive homeostatic response, repeated loading induces lesions and subchondral changes that are similar to OA [76]. This model allows for the study of long-term effects of injury and can be useful for regenerative techniques that target patients who have had OA develop from chronic injury overuse.

In a model that is similar to cyclic AC tibial compression, tibial compression overload applies a single cycle with a load of 12 N and a speed of 500 mm/s to produce a severe and immediate injury with subsequent midsubstance rupture of the ACL in mice up to 8 weeks old. Alternately, an avulsion fracture of the ACL from the underlying bone may also occur at lower speeds [75, 81–83]. Similar to the ACLT model, a ruptured ACL leads to instability of the entire joint and increased anterior translocation of the tibia in relation to the femur. This alters the knee biomechanics, inducing apoptosis of cells and erosion of articular cartilage with extension to the subchondral bone. Studies have also revealed an increased concentration of inflammatory cytokines, and hemarthrosis as a result of injury leads to rapid synovial inflammation and synovial cell proliferation as early as 2 weeks post-injury. These changes lead to the formation of visible ectopic cartilaginous nodules or neocartilage metaplasia [83]. Due to the acute changes and nature of the injury, this model has advantages in studying low-energy sports injuries and regenerative therapies following acute injuries. Efficacy in long-term studies is still in question due to the formation of severe osteophytes as compensation for long-term joint instability [44].

Transarticular impact, as a noninvasive model, has also been used in certain dog and rabbit species. The transarticular impact model utilizes a dropping tower with an approximate load of 2000 N causing an impact on the patellofemoral joint of the immobile, flexed knee, without breaking the skin. Some models utilize a pendulum swing to replicate trauma to the femoral condyles as well [84–87]. The subfracture impact leads to osteochondral lesions within a year of the injury. Traditionally, this model has aided research in adverse changes and articular healing following the impact [18].

Given that the dynamic models do not require any form of surgical intervention or sterile environments, the risk of infection or inflammation is reduced when compared to surgical OA models. In addition, dynamic models are powered by machines to create a mechanical insult and are less dependent on the surgical skills of the researcher, thus, more consistent results can be easily produced [18]. In addition, PTOA occurs after an external injury to the knee as opposed to surgical intervention, allowing for replication of similar biomechanics in human injury [18]. However, the cost of the machinery used in dynamic models and calibration and maintenance may outweigh the benefits provided.

### Synthetic Animal Models

Synthetic or chemically induced animal models utilize a chemical reaction to induce OA. An inflammatory or toxic compound is directly injected into the joint to produce osteoarthritic lesions which compromise joint integrity and function [52]. One of the first compounds that were used in synthetic models was papain. Papain is a proteolytic enzyme that degrades proteoglycans in cartilage, resulting in the release of chondroitin sulfate from the matrix [52]. Proteoglycans provide cartilage with compressive resistance through water absorption and are thus an essential component for structure and stability [12]. Mice usually develop lesions within 3 weeks of intra-articular injection. OA has also been successfully induced in other species such as rats and rabbits. However, the use of papain has become less common, as more effective chemical models have emerged [52, 88, 89].

Mono-iodoacetate (MIA) is becoming increasingly popular in recent studies for the synthetic induction of OA in mice and rat models. MIA interferes with cartilage metabolism by reducing glyceraldehydes-3 phosphate dehydrogenase activity in chondrocytes [52]. Cartilage depends on anaerobic metabolism due to its avascular nature, and inhibition of this enzyme leads to a decrease of available intracellular adenosine triphosphate (ATP). This results in chondrocyte death, osteophyte formation, and articular cartilage degradation [90, 91]. Systemic administration of quinolone antibiotics has well-documented side effects that include arthropathy and tendinopathy, especially when given during the growth phase of animals. Quinolones given to immature dogs and guinea pigs lead to gait disorders and irreversible losses of proteoglycans, chondrocytes, and extracellular matrix. Enrofloxacin, in particular, is the most broadly used quinolone antibiotic during animal experiments for OA induction [18, 42].

**Fig. 5:**
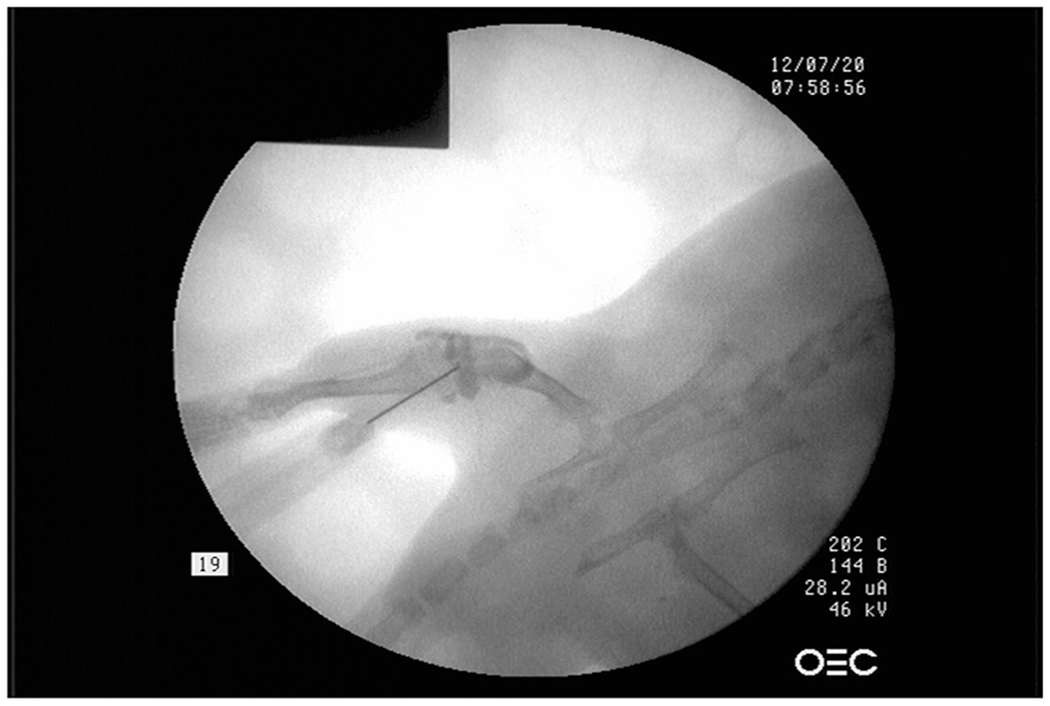
C-arm radiograph of a guided injection of intra-articular collagenase in the knee joint of a mouse to induce osteoarthritic changes. Collagenase is encapsulated within the joint space

Intra-articular injection of collagenase has also gained popularity among animal studies due to its ease of preparation and low cost (Fig. 5). Collagenase degrades type I collagen fibers, one of the main components of articular cartilage, which reduces the collagen matrix in the tendons and ligaments within the articular space [92, 93]. Usually given as two doses of either 250 U or 500 U with the second dose applied 3–5 days after the initial injection with lesions appearing up to 3 weeks after injection [42]. The reaction observed within the cartilage and corresponding joint instability is reported to closely resemble the changes seen in human OA [94]. In addition, collagenase has been shown to induce local tissue damage on articular cartilage without any adverse or degenerative effects in other tissues [95].

Collagenase-induced OA models have been featured in a few regenerative engineering studies focused on stem cell therapy. Ter Huurne et al. investigated the anti-inflammatory and chondroprotective effects of ADSCs in mice with collagenase-induced OA. ADSCs demonstrated inhibition of synovial lining thickening and cartilage destruction and demonstrated chondroprotective effects against joint destruction through both anabolic and catabolic mediators [96]. Another study utilizing a similar collagenase-induced OA model in mice showed that OA responded to treatment with bone marrow-derived mesenchymal stem cells (BMSC) as chondrocyte homeostasis was re-established and inflammation in the joint was relieved [97]. As a well-established OA model, collagenase can also be used to create a reliable OA control for comparing the efficacy of studies involving regenerative engineering therapeutics as intervention [97].

MIA-induced OA rat models have been recently used to investigate the efficacy of human amniotic fluid stem cells (AFSC) due to their ability to produce exosomes with growth factors and immunomodulating molecules [98]. Zavatti et al. compared treatment between AFSCs and commercial exosomes with typical markers of hepatocyte growth factor (HGF), TGF- β, and indoleamine 2,3-dioxygenase (IDO). Exosome-treated groups showed enhanced histological scores by week 3, and TGF-β-rich exosome samples displayed an almost complete restoration of cartilage [99].

The largest drawback of synthetic models is that the pathophysiology of OA induction differs greatly from OA in humans since the mechanism is based on the injected compound, while in humans, the disease progression is usually a result of chronic, degenerative changes or PTOA [52]. Nonetheless, synthetic animal models are relatively inexpensive and quickly inducible, making them advantageous for short-term studies.

## Tertiary Osteoarthritic Animal Models

The tertiary OA model aims to exploit the degeneration of the knee or stifle joint after surgical resection of tissue and the use of a controlled exercise regimen to stimulate OA progression post-surgery. The combination of a secondary model with a regimen to accelerate disease may produce a more reflective in vivo animal model of patients who develop OA due to a traumatic joint injury followed by continuous overuse of the joint. It is hypothesized that this model may be used by investigators to monitor the progression of OA following overuse after trauma due to sports or military injuries without sufficient treatment or recovery. In addition, minor forms of trauma have the potential to go undetected due to their asymptomatic nature, and continuous overuse may result in the development of degenerative joint disease. This model differs slightly from PTOA models as the primary focus is not on just providing a gross lesion that will eventually lead to degenerative changes but also on the effects of overuse and motion once an injury has been sustained.

**Fig. 6:**
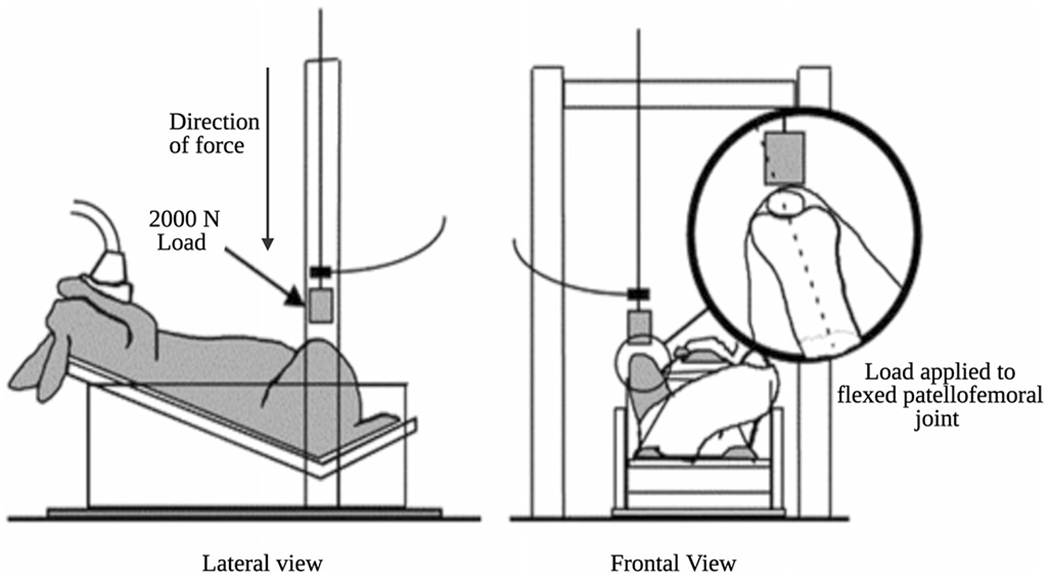
Dynamic/noninvasive model of OA: transarticular impact. A mass of 2000N with a padded interface is dropped onto the flexed patellofemoral joint to produce intra-articular changes within the knee joint (Ewers et al. [87])

One of the earliest studies that incorporated the tertiary OA model was designed by Newberry et al. to investigate the effect of blunt-impact trauma on the knee followed by physical exercise. A blunt impact was delivered to the patellofemoral joint in mature Flemish Giant rabbits, similar to the transarticular impact model described in the previous section (Fig. 6). However, a fracture was not produced in these animals. Following the injury, the rabbits received daily exercise consisting of 10 min of running at 0.3 mph on a treadmill. Upon histological and biomechanical analyses of joint tissue, degenerative changes were observed at various time points. In addition, the results of this study recommended that the contralateral limb should not be used as a control [100]. Murphy et al. attempted this model in a larger animal, a goat, and found that a combination of surgical resection of the ACL and medial meniscus with overuse of the traumatized joint leads to acute OA [101]. Murphy et al. also integrated stem cell therapy in the form of BMSC in their study, which demonstrated regeneration of the meniscal tissue [101].

More recently, Ude et al. conducted a study to investigate the regenerative capacity of ADSCs and BMSCs in an OA model that was induced by a combination of surgery and motion [102]. Complete resection of the ACL and medial meniscus was performed in the right knee of adult male sheep, which resulted in osteoarthritic changes in both the medial and lateral compartments, but the medial compartment was more severely affected as a result of the removal of the meniscus. Following a 3-week recovery period from surgery, an exercise regime of 100 m daily was implemented for 3 weeks. The arthroscopic evaluation of post OA induction in the control group revealed various degenerative and inflammatory changes including focal lesions to medial femoral condyle, medial tibial plateau, and patella-femoral groove. Grossly, the groups treated with ADSCS and BMSCs showed regeneration of de novo cartilages and menisci within 6 weeks of treatment [102]. However, there was no significant difference in regenerative outcomes between ADSCS and BMSCs, indicating that both are promising options for cartilage regenerative therapies [102].

## Concluding Remarks and Future Directions

Animal models of OA play a large role in the investigation of therapeutic techniques that employ tissue regeneration strategies by providing a preclinical model of disease. However, OA is a multifactorial disease, making it difficult to be modeled in a single animal species and technique. Each model of inducing osteoarthritic changes in animals has many associated advantages and disadvantages based on the method of induction and the animal of choice. Mice are highly utilized in animal models due to their low cost, relative ease of handling, and genetic manipulation, making them more suitable for synthetic and genetically related primary OA models [46]. However, load biomechanics and thin cartilage in mice models may become problematic due to the extremely small size when compared to humans. Load biomechanics are particularly important in OA studies, and the thin cartilage in mice makes it difficult to induce small defects that may progress to OA, making surgically induced OA procedures a challenge [46]. On the other hand, rats have much thicker cartilage than mice which makes it possible to induce both partial- and full-thickness cartilage defects [46].

Other larger animal models such as goats, sheep, and horses have much larger knee joints, with a comparable size to humans, which allows for evaluation via arthroscopy and magnetic resonance imaging (MRI). However, unlike mice and rats, larger animals are not as prone to develop spontaneous OA as rapidly and usually require surgical or dynamic induction to produce osteoarthritic lesions [46]. Large animal models also share heterogeneity with humans and the genetic, physiological, and complex interactions with the environment, making them ideal for evaluating the safety and efficacy of new therapies. Furthermore, large animal models that are similar in size and weight to a human may be crucial for implantations and more appropriate for biomechanical studies. Thus, choosing the ideal animal model to answer the proposed scientific question is a top priority in securing a successful outcome [103].

As the field of regenerative engineering continues to grow, recent advances in advanced biomaterials, nanotechnology, stem cell science, and developmental biology have provided researchers and engineers with alternative approaches to repair tissue and organ systems [3, 104–109]. The concept of convergence provides the potential to develop novel treatments and revolutionize therapeutic approaches to musculoskeletal conditions. However, it is important to note that key challenges still exist, especially with achieving product consistency and efficacy [110, 111]. Nonetheless, as more research is done within the field, solutions and strategies to overcome these issues will be further explored. With this framework in place, regenerative engineering has the potential for not only the regeneration of native tissue in musculoskeletal conditions such as OA but the regeneration of complex tissues and organ systems as well.
